# Immunotherapy strategies and prospects for acute lung injury: Focus on immune cells and cytokines

**DOI:** 10.3389/fphar.2022.1103309

**Published:** 2022-12-22

**Authors:** Wenfang Zhu, Yiwen Zhang, Yinghong Wang

**Affiliations:** ^1^ Department of Respiratory Medicine, Anhui Chest Hospital, Hefei, China; ^2^ Department of Pharmacy, Anhui Provincial Cancer Hospital, The First Affiliated Hospital of USTC, Division of Life Sciences and Medicine, University of Science and Technology of China, Hefei, China

**Keywords:** immunotherapy, acute lung injury, immune cells, cytokines, prospects

## Abstract

Acute lung injury/acute respiratory distress syndrome (ALI/ARDS) is a disastrous condition, which can be caused by a wide range of diseases, such as pneumonia, sepsis, traumas, and the most recent, COVID-19. Even though we have gained an improved understanding of acute lung injury/acute respiratory distress syndrome pathogenesis and treatment mechanism, there is still no effective treatment for acute lung injury/acute respiratory distress syndrome, which is partly responsible for the unacceptable mortality rate. In the pathogenesis of acute lung injury, the inflammatory storm is the main pathological feature. More and more evidences show that immune cells and cytokines secreted by immune cells play an irreplaceable role in the pathogenesis of acute lung injury. Therefore, here we mainly reviewed the role of various immune cells in acute lung injury from the perspective of immunotherapy, and elaborated the crosstalk of immune cells and cytokines, aiming to provide novel ideas and targets for the treatment of acute lung injury.

## 1 Introduction

Acute lung injury (ALI) is a clinical syndrome involved in inflammation and enhanced pulmonary capillary permeability, which can cause ARDS in severe cases. Its pathological characteristics are diffuse alveolar capillary membrane injury (Perl et al., 2011). Acute lung injury/acute respiratory distress syndrome (ALI/ARDS) encompasses a wide range of pathologic processes, including multiple organ dysfunction syndrome, which has a 40% mortality rate (Matthay et al., 2019). Currently, COVID-19 patients die primarily from ALI/ARDS. There are reports that about 1/3 of hospitalized COVID-19 patients suffered from ARDS, with a frightening 70% mortality rate in these cases (COVID-19/ARDS) (Tzotzos et al., 2020). So far, severe COVID-19 infection has brought millions of people deaths globally. More than 90% of death victims of COVID-19 died from ARDS, suggesting that the majority of deaths caused by COVID-19 are related to ARDS (Tzotzos et al., 2020).

Currently, major efforts are being made to discover mechanism for cognizing and ameliorating ALI, and there has been extensive coverage of these topics in recent reviews (Butt et al., 2016b; Liu et al., 2022; Vichare and Janjic, 2022). Here, however, we focus more on recent developments regarding the role of immunotherapy to ALI/ADRS. We succinctly recapitulate the current insights into ALI signaling, indicate the molecular mechanisms that contribute to immune pathway activation in a variety of pathophysiological situations, and discussed its role in disease-related preclinical models. In light of recent advances in understanding of the ALI pathology and treatment, we explore possible pharmacological intervention strategies focused on these immune cells and discuss their therapeutic potential in treating inflammation associated with ALI.

## 2 Etiology and pathology of ALI

ALI is presently recognized as an acute diffuse lung injury that can be caused directly through the airway (e.g., inhalation of human stomach contents or toxic substances) or indirectly through the bloodstream (e.g., sepsis or trauma) (Ware and Matthay, 2000). The etiology of ALI is quite numerous, and it can be caused by either internal or external pathogens, including severe infections, sepsis, trauma, shock, acute pancreatitis, iatrogenic lung damage caused by radiotherapy and chemotherapy, inhalation of harmful substances, etc., (Thompson et al., 2017). From the clinical point of view, it can be divided into 10 categories, such as shock, trauma, severe infection and sepsis, aspiration of harmful fluids, inhalation of damaging gases, drugs and metabolic diseases. No matter which etiology causes ALI, its pathogenesis is related to out-of-control inflammatory response. The most common reason of ALI is indirect lung injury, for instance sepsis, trauma and blood transfusion. These triggers can travel through the bloodstream to the lungs and throughout the body, causing systemic inflammatory responses (Castro, 2006; Butt et al., 2016a).

Diffuse alveolar injury is the dominant pathological feature of ALI (Castro, 2006). Moreover, pathological features of ALI also include uncontrolled inflammatory response during neutrophil movement, the production and secretion of proinflammatory cytokines, large numbers of lung epithelial cells apoptosis, loss of alveolar integrity, the damage of alveolar capillary membrane and barrier functions. The flow of protein edema fluid into the alveoli can lead to the inactivation of surfactants and the loss of the protective layer on the alveolar surface, thus destroying the surface cell structure (Mokrá, 2020). Moreover, an injured capillary endothelium attaches neutrophils, which pass through the interstitial cavity to the alveolar cavity filled with protein-rich edema fluid (Scozzi et al., 2022). ALI is also accompanied by local or distant inflammation, and the extent of lung damage depends on whether it is directly to the lungs or caused by external factors, such as lipopolysaccharides (LPS) or inflammatory mediators produced by other organs that circulate throughout the body to the lungs (Liu et al., 2015).

Inflammation in ALI can be triggered through both exogenous and endogenous pathways. Exogenous pathways like bacterial antigens rouse inflammatory responses by triggering toll-like receptors (TLR). Chemical damage can also induce cell membrane damage and oxidative stress, resulting in activation of various intracellular kinases (Arora et al., 2019). The endogenous pathways are mainly composed of hazard signal molecules, members of the damage-associated molecular pattern (DAMP) released by dead cells or local inflammatory cells, which engage and recruit immune cells by binding to various receptors, including TLRs and IL-1 receptors (IL-1R), as a result, the pro-inflammatory pathway is activated (Tolle and Standiford, 2013). At the early stage of ALI, the damage of alveolar-capillary barrier and the formation of pulmonary edema are the main pathological features of ALI. Studies have shown that the change of alveolar barrier function is closely related to inflammation, and pathological results show that when ALI patients suffer from the onset of disease, there may be a great deal of white blood cells in the lungs, alveolar edema, bleeding and other manifestations (Herold et al., 2013). Ultrastructural studies of the lungs in patients with sepsis secondary to ALI have shown significant increases in the number of intravascular and extravascular neutrophils (PMN), platelets, and fibrin, and endothelial and epithelial lesions, which are considered to be inflammatory edema of ALI (Maniatis et al., 2008).

## 3 Review of current treatment strategies for ALI

According to the pathogenesis of ALI/ARDS, the current drugs used to treat ALI/ARDS mainly include the following categories.

### 3.1 Vasodilator drugs

Nitric oxide (NO) can induce pulmonary vasodilation without systemic vasodilation through inhalation of NO. Although NO can improve oxygen levels in some extent, many studies have shown that it could not reduce the mortality of ALI/ARDS, and might also cause complications such as pulmonary edema and pumonary hypertension. Therefore, it is not routinely recommended for clinical use. Prostaglandin is another drug for vasodilating, which has the same effect as NO. It is convenient to administer by aerosol inhalation and can be used as a substitute for NO, but its disadvantage is that it is controversial and expensive (Adhikari et al., 2007; White et al., 2008; Afshari et al., 2010; Bosch et al., 2022).

### 3.2 Surfactant (pulmonary surfactant)

Surfactant is mainly composed of lipids and related proteins, can maintain the structural stability of alveoli by reducing the surface tension of alveoli, and can also prevent pulmonary edema and reduce inflammation (Lewis and Veldhuizen, 2006; Lan et al., 2021). However, due to the poor therapeutic effect of pulmonary surfactant, the dosage and usage are still controversial, so it should be used cautiously (Ali et al., 2021).

### 3.3 Antioxidants

Because immune cells produce a large number of free radicals during the inflammatory reaction of ALI, a therapeutic method that uses antioxidants to combat free radicals is developed (Sarma and Ward, 2011). Such as glutathione (Moradi et al., 2009), vitamin C (Fowler et al., 2019; Holford et al., 2020), and vitamin E (Wang Y. et al., 2022), but their effects are still to be studied.

### 3.4 β2 receptor agonist

It is controversial to use an agonist for the treatment of ALI and ARDS patients, in some studies and clinical datas, results showed that β2 agonists have no benefit in improving survival, but can increase morbidity. β2 agonists are not recommended for ALI/ARDS patients based on current evidence (Singh et al., 2014).

### 3.5 Anti-inflammatory drugs

Corticosteroids are mainly used, however, there are a number of studies that suggest that early use of high-dose corticosteroids can increase the death of ALI/ARDS patients, so the use is not recommended (Mokra et al., 2019; Bobot et al., 2022).

### 3.6 Stem cell therapy

ALI has been treated using mesenchymal stem cells (MSCs), which can modulate interconnected signal pathways including PI3K/AKT, Wnt, and NF-κB to alleviate inflammation. There are MSCs in various tissues that are capable of self-renewal and differentiation. Their activation is triggered by specific substances or environments, and they can be directed to damaged tissues, where they regenerate and repair the damage. It has been shown that exosomes, as well as cytokines involved in the paracrine pathway of MSCs, are effective in treating ALI (Fernández-Francos et al., 2021; Wang et al., 2022d).

We summarized the advantages and disadvantages of previous ALI treatment methods in the Table 1. According to the above treatment methods, it is currently impossible to find a gratifying drug that can effectively treat ALI/ARDS. In light of this, further research on the pathogenesis of ALI/ARDS is urgently needed, as well as the identification of more effective therapeutic methods and targets.

**TABLE 1:** Advantages and disadvantages of existing acute lung injury (ALI) treatment strategies.

Strategies	Representative	Advantages	Disadvantages
Vasodilator drugs	NO	Convenient and Effective	Short duration. No reduction in case fatality
	Prostaglandin	Convenient	Expensive, Controversial
Surfactant	Pulmonary Surfactant	Effective	Controversy usage
Antioxidants	Glutathione, Vitamin C/E	Cheap	Low effective
β2 receptor agonist	Propranolol Metoprolol	Effective	Allergies. Arrhythmia, Heart failure
Anti-inflammatory drugs	Corticosteroids	Effective	Increase the mortality of late ARDS
Stem cell therapy	Mesenchymal stem cells	Effective	Expensive

## 4 The immune cells in ALI/ARDS

### 4.1 Neutrophil

Neutrophils, as an immune system cell, can circulate freely inside blood vessels and can be recruited to inflammatory sites when a microbial infection occurs in the human body. It is thought that neutrophil activation and recruitment play a major role in ALI/ARDS progression. Among the first cells to be recruited to inflammation sites, neutrophils have powerful antimicrobial properties, including oxidants, proteinases and cationic peptides. As early immunologic effectors in ALI, neutrophils could stimulate the expression of NF-κB, IL-1β, macrophage inflammatory protein-2 (MIP-2), and tumor necrosis factor-a (TNF-a) (Abraham et al., 2000; Yang et al., 2003). Microbicidal compounds, however, can paradoxically damage host tissues under pathological circumstances (Grommes and Soehnlein, 2011). Neutrophils are known to bind to each other *via* neutrophil extracellular traps (NETs). This process is called netosis, and it is a specific type of cell death, different from necrosis and apoptosis (Zhu et al., 2022). The formation of NETs takes place when neutrophils are exposed to bacteria, fungi, activated platelets, or numerous inflammatory stimuli, and this process is affiliated with dramatic changes in the morphology of the cells (Meyers et al., 2022). It is DNA and granular antimicrobial proteins that determine NETs’ antimicrobial properties. Both oxidative and non-oxidative mechanisms are used in the killing of pathogens trapped in NETs (Mamtimin et al., 2022). In addition, it has also been reported that chromatin and proteases released when NETs form can affect procoagulant and prothrombotic factors and participate in the formation of blood clots (Meyers et al., 2022).

There has also been evidence of NETs in patients with ALI/ARDS, where they appear to participate in chronic inflammation processes. Poor degradation and excessive NET formation, however, can exacerbate immune responses and tissue damage. Through the promotion of macrophage polarization to the M1 phenotype, NETs can facilitate ARDS inflammation in the process of the acute phase of the disease (Song et al., 2019). Furthermore, in mice with ALI induced by LPS, NETs formed, caused organ damage, and induced an inflammatory response. Degradation of NETs by DNase I contributed to NET protein clearance and protected against ALI (Liu et al., 2016). In addition, neutrophils are involved in the formation of blood clots. Neutrophils adhered better to activated platelets due to increased ICAM-1 expression in the endothelial cells. As a result of inhibiting platelet-neutrophil aggregation, gas exchange was improved, neutrophil recruitment was reduced, and neutrophil permeability was reduced. These main outcomes were confirmed in a sepsis-induced model of ALI (Zarbock et al., 2006). Among those with sepsis-induced acute lower respiratory infection, Park et al. (2019) noted a rapid decline in the functional capillary ratio during the early stages. By capturing images intravitally, this decrease was attributed to the generation of dead space, caused by prolonged neutrophil entrapment in capillaries. Their results further indicated that neutrophils also displayed an arrest-like dynamic behavior and an extended sequestration time, which sparked neutrophil aggregates inside capillaries and arterioles. As a result of septic shock, osteopontin’s neutralization could lessen neutrophil migration into the lungs (Hirano et al., 2015). In addition, p38δ and PKD1 oppositely modulate PTEN activity in neutrophils, therefore, they will be able to control their extravasation and chemotaxis. PKD1 phosphorylates p85δ to promote its relation with PTEN, resulting in polarized PTEN activity, consequently regulating neutrophil migration (Ittner et al., 2012).

### 4.2 Alveolar macrophages

Although it is well known that neutrophil influx and activation within the lungs participate in ALI pathogenesis, there is increasing evidence that alveolar macrophages (AM) are also involved in modulating inflammatory responses. Macrophages are classified into classical (M1) and alternative (M2) macrophages based on their roles in host defense, despite these distinct *in vitro* classifications, macrophage polarization probably exists in a continuum (Ruytinx et al., 2018; Orecchioni et al., 2019). M1 cells produce high levels of proinflammatory factors such as IL-1β, IL-12, TNF-α, and inducible nitric oxide synthase (iNOS), which are induced by Th1 cytokines. Th2 cytokines, such as IL-4 and IL-13, are known to induce the M2 phenotype, which is characterized by the production of anti-inflammatory molecules such as IL-10 (Mantovani et al., 2002; Mantovani et al., 2005). It is important to note that macrophages are unlike other discrete leukocyte populations in that they retain their plasticity and can be altered by various factors within the microenvironment, including cytokine milieus, among others (Johnston et al., 2012). This study suggested that reprogramming macrophages may contribute to ALI progression.

In ALI, there are a variety of factors that can induce macrophages to change to M1 type, such as LPS and ischemia-reperfusion injury (Zhao et al., 2006; Jiang et al., 2022). There is increasing evidence that inhibiting the transformation of macrophages in various ways can effectively alleviate ALI and even the disease course of COVID-19 (Wang et al., 2022c). Current studies have shown that macrophages are involved in ALI inflammation with two modes, one is that they are induced to secrete inflammatory factors. According to this study, recruited lung macrophages inhibit IL-1β–mediated ALI in gram-negative pneumonia by release of IL-1 receptor antagonist (Herold et al., 2011). Another mode of action is interaction with neutrophils. As is well known that early in ALI there is a penetration of neutrophil, upon intratracheal LPS administration, necrotic AM released pro-interleukin-1α(IL-1α), which activated endothelial cells (EC) to initiate vascular leakage by losing VE-cadherin. Ultimately, it can promote the infiltration of neutrophils and promote the development of ALI (Dagvadorj et al., 2015). In addition, by regulating NF-kB activity and inducing M1 macrophage polarization, exosomal miR-30d-5p from PMNs can influence sepsis-related ALI (Jiao et al., 2021). In conclusion, the crosstalk between AM and neutrophils deserves more research.

### 4.3 T cells

According to current theories, ARDS is caused by the destruction of alveolar endothelial and epithelial tissue by platelet-derived products and innate immune cells. As of yet, it is unclear what role adaptive immune cells play in ARDS. In terms of immunological function, T cells can be divided into T helper cells (Th), Cytotoxic T cell and Regulatory T cell (Treg). CD8^+^T cells are killer T cells, which can release cytokines and perforin to kill virus-infected cells or tumor cells when activated. There is evidence that a malaria-associated lung injury requires an overreaction of CD8^+^T cells. Excited CD8^+^T cells migrate to the lungs during infection, and immune-mediated anti-CD8 antibody treatment ameliorates pulmonary damage (Claser et al., 2019).

There is evidence that regulatory T-cells (Tregs) facilitate tissue repair and promote ARDS resolution. It has also been shown that Treg-depleted mice have impaired Th1 and Th17 immune responses, suggesting that Tregs are indispensable for tissue repair, modulating and promoting the Th immune response in LPS-induced pulmonary inflammation (Tan et al., 2019). Normally, T-regs are found in lymphoid tissues and peripheral blood, Leukotrienes B4 (LTB4) can recruit CD4^+^CD25^+^ Foxp3^+^ Regulatory T Cells during ALI, which reduce the inflammation of the ALI (D'Alessio et al., 2009; Wang et al., 2012). In general, Treg cells communicate with other T cells by secreting anti-inflammatory factors. For example, the production of IL-10 by Treg cells and dendritic cells can protect the lungs from injury caused by transfusion (Kapur et al., 2017). In addition, it is noteworthy that Treg can also reduce the proliferation of ALI fibers by reducing fibrocyte recruitment (Garibaldi et al., 2013). It has also been reported that CD39^+^ Treg cells reduce LPS induced ALI through autophagy and ERK/FOS pathways (Chen et al., 2020).

Recent studies have focused on the effect and function of Treg on ALI, but further studies on Th cells are needed. Compared with Treg, Th cells may have a more unique function, and it has been reported that CTLA4 plays a role in T cell pathways in ALI models (Nakajima et al., 2010). Importantly, a gradual understanding of Th17’s function is emerging in ALI, Th17 cells are T helper cells that secrete a distinct subset of T cell cytokines, including IL-17A and F, IL-21 and IL-22. IL-17A and F, especially, are released in the setting of bacterial infections and have distinctive roles in reply to bacterial and fungal pathogens (Li et al., 2015; Sakaguchi et al., 2016).

### 4.4 Monocytes

Monocytes originate from hematopoietic stem cells in the bone marrow and develop in the bone marrow. When they enter the blood, they are still immature cells. Monocytes also take part in the immune response, after phagocytosis of antigen to carry the antigen epitope to the lymphocyte, inducing lymphocyte specific immune response (Merad and Martin, 2020).

Monocytes in the lung can be divided into mature and immature mononuclear cells. The plasticity of immature mononuclear cells in diseases is higher. Studies have indicated that immature monocytes promote cardiopulmonary bypass-induced ALI by generating inflammatory descendants (Xing et al., 2017). The reason is that immature CD14^low^ CD16− monocytes have a limited ability to produce TNF-α and inhibit T cell proliferation mediated by T cell receptor signaling. However, these immature cells are highly proliferative and can differentiate into mature CD14^high^ CD16^+^ monocytes that produce TNF-α (Xing et al., 2017). Crosstalk between monocytes and other immune cells has always been the focus of research. It is believed that the communication between monocytes and immune cells is a key point. In ALI, monocytes are activated by LPS and can recruit neutrophils to the site of inflammation, thus achieving a synergistic effect (Dhaliwal et al., 2012). In addition, Plasmacytoid Dendritic Cells can recruit monocytes to activate inflammatory in mice lung injury (Venet et al., 2010). Interestingly, it is not only lung queued monocytes that have a direct effect. Chemokines secreted by monocytes in blood play a pivotal role in the progression and outcome of lung injury during ischemia-reperfusion ALI. The main reason is that chemokines in the blood can activate lung monocytes and induce inflammatory expression after reaching the lungs. Blocking this process can significantly reduce the extent of lung damage (McKenzie et al., 2014).

### 4.5 Other immune cells

**FIGURE 1:**
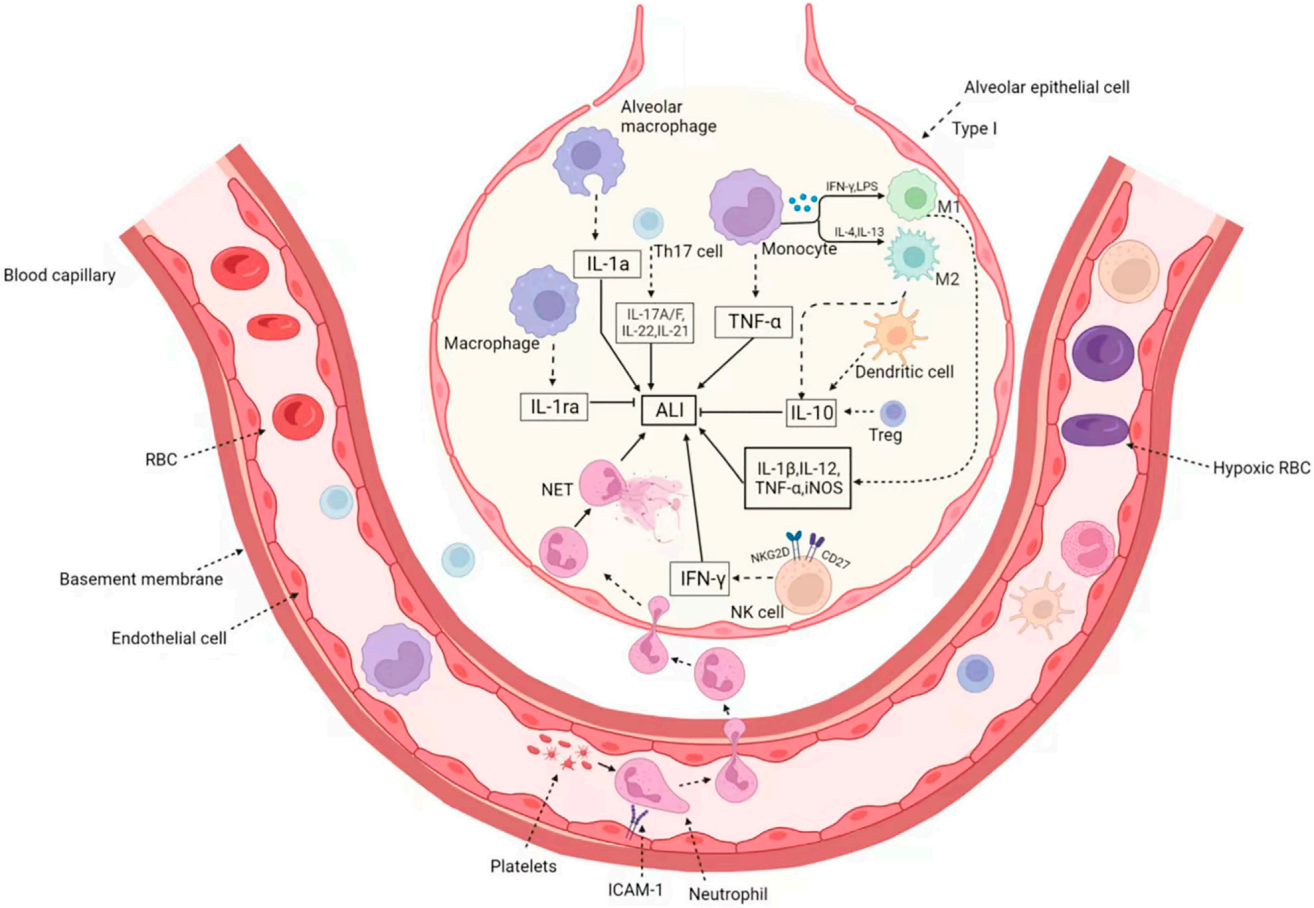
The immune cells in Acute lung injury/acute respiratory distress syndrome. When ALI occurs, various immune cells are recruited through the blood vessels to the inflammatory region and enter the alveoli through the swollen outer walls of the blood vessels. On the one hand, neutrophils recruit platelets to form microthrombus, endothelial cells expressed ICAM-1 and increased platelet adhesion. On the other hand, they form NET through capillaries to enlarge inflammatory lesions. The activated macrophages are divided into M1 type and M2 type. M1-type macrophages induced by IFN-γ and LPS secrete pro-inflammatory factors such as IL-1β, IL-12, TNF-α, and iNOS. While M1 macrophages induced by IL-4 and IL-13 have the opposite function, which product IL-10 and relief ALI. In addition, eosinophils, NK cells, dendritic cell and Treg cells were also recruited to play different roles. Treg and dendritic cells secrete IL-10 to inhibit inflammatory response, and activated NK cells overexpress NKG2D and CD27 receptors, release IFN-γ, and promote the occurrence of ALI.

In addition, there are some studies on NK cells and eosinophils in ALI. Eosinophils, like other granulocytes, are derived from blood-forming stem cells in the bone marrow. Eosinophils have the function of killing bacteria and parasites, and are very important cells in immune response and allergic reaction. Eosinophils can release the contents of the particles, causing tissue damage and promoting the progression of inflammation (Grisaru-Tal et al., 2022). Recent studies have shown that eosinophils have an excellent protective effect against ALI, while eosinopenia may increase mortality risks. Apart from pulmonary macrophages, homeostasis eosinophils are a newly discovered cell group, which play a key role in the ALI. The LPS challenge induces rapid agglomeration of eosinophils from the peripheral circulation into the lungs. Loss of eosinophils increases LPS-induced neutrophilic inflammation and eventual injury levels. Homeostasis CD101^−^ but not allergic CD101^+^ eosinophils play an anti-inflammatory role in ALI (Zhu et al., 2020). In addition, IL-33-induced eosinophilia is critical for preventing death induced by *staphylococcus aureus* (Krishack et al., 2021), which provides evidence for novel and potentially beneficial effects of eosinophils. As a powerful toxic cell, NK cells play a crucial role in a variety of diseases. As the first line of defense, NK cells are key players in innate immunity. However, its function in ALI is seriously underestimated. Activated lung NK cells overexpress activated receptors NKG2D and CD27, and become functional NK cells by producing large amounts of interferon *γ*, which is the cause of acute lung immune injury. The decrease of NK cells significantly reduced lung immune damage, total inflammatory cell infiltration and IFN-γ production in bronchoalveolar lavage fluid (BALF) (Li et al., 2012). We have sorted out the role and mechanism of immune cells in ALI as shown in the Figure 1.

## 5 The immune-related factors in ALI/ARDS

### 5.1 Interleukin

There are several functions of interleukins in inflammation, including transmitting information, activating and regulating immune cells, and promoting proliferation and differentiation of T and B cells (Briukhovetska et al., 2021). In ALI, IL-1β is at the top of the list. As one of the most common pro-inflammatory factors, numerous studies have focused on the anti-IL-1β function. Recruitment of neutrophils is known to be critical in prophase ALI. Studies have reported that Il-1β is also involved in this process. In lung injury after lung transplantation, classical monocytes infiltrated by the graft produce myD88-dependent IL-1β, thus mediating neutrophil exosmosis (Hsiao et al., 2018). In addition, IL-1β in acid—induced and sepsis—induced ALI is also associated with the development of inflammation (Mizushina et al., 2019; Xiong et al., 2020). So, the source of IL-1β has been a focus of attention, on the one hand, IL-1β is produced by different immune cells, such as alveolar macrophages (Xu et al., 2022), monocytes (Hsiao et al., 2018). On the other hand, NLRP3 inflammasome also mediates the shearing and maturation of IL-1β (Mizushina et al., 2019).

Another family of cytokines that play a pro-inflammatory role in ALI is IL-17. Studies have shown that IL-17 is primarily congenital lymphocyte production in ALI (Muir et al., 2016), in the case of LPS-induced airway epithelial cell injury, the interaction between IL-17A and endoplasmic reticulum stress is pivotal and exhibits positive feedback (Kim et al., 2015). In addition, the production of IL-17 by lung γδ T cells is also considered to be one of the important factors in the pathogenesis of ALI (Menoret et al., 2018).

In addition, several other interleukins are associated with the progression of ALI. For example, blocking IL-3 and IL-5 can significantly reduce the hyperinflammatory response in ARDS models (Wang H. et al., 2022). Of note is the self-sustaining IL-8 cycle driving the prethrombotic neutrophil phenotype in severe COVID-19 (Kaiser et al., 2021). Coincidentally, interleukin-36γ and IL-36 receptor signaling pathways reconcile damaged host immunity and lung damage in cytotoxic *Pseudomonas aeruginosa* lung infection (Aoyagi et al., 2017).

Conversely, some studies have reported on the anti-inflammatory interleukin. For example, IL-10 and IL-35 have a protective effect on ARDS by increasing CD4+Treg ratio in extrapulmonary ARDS (Kapur et al., 2017; Wang et al., 2019). ARDS also can be alleviated by IL-4 mediated reprogramming of lung macrophages (D'Alessio et al., 2016). In addition, IL-33-dependent regulatory T cell accumulation mediates lung epithelial regeneration at the end stage of ALI (Tan et al., 2021).

### 5.2 Interferon

Besides interleukin, interferon also plays an irreplaceable role in ALI. The function of interferon in ALI is two-sided. On the one hand, influenza-induced IFN-γ triggers the hyperreactivity of bone marrow cells to MRSA, resulting in excessive inflammatory responses and fatal lung injury during co-infection (Verma et al., 2022). On the other hand, IFN-β can restore the function of damaged alveolar macrophages by recruiting neutrophils to the alveoli, thus improving the survival rate (Hiruma et al., 2018; Sekheri et al., 2022).

### 5.3 NLRP3 inflammasome

In addition to membrane-bound Pattern Recognition Receptors (PRRs), cytoplasmic PRRs, such as the NLRP3 inflammasome, indirectly recognize PAMP and DAMP. This process can be called a molecular process that detects changes in homeostasis. The NLRP3 inflammasome consists of NLRP3, ASC and caspase-1. When NLRP3 inflammasome recognizes PAMP and DAMP, caspase-1 splits IL-1β and IL-18, producing active IL-1β and IL-18, which mediate multiple inflammatory responses (Harapas et al., 2022). The role of NLRP3 in ALI has been controversial. Studies have shown that macrophages are over-activated in LPS-induced ALI, activating NLRP3 inflammasome and triggering pyroptosis, a process that exacerbates the disease of ALI (Li et al., 2018; Hu et al., 2022). In addition, NLRP3-mediated pyroptosis may not be limited to macrophages, but may also be discovered in neutrophils (Feng et al., 2017). In brief, the role of the inflammasome represented by NLRP3 in ALI deserves continuous attention.

### 5.4 Other immune-related factors

The role of most immune-related factors is reflected in the understanding of immune cell function. The regulation of alveolar macrophages is particularly important in ALI. It is well known that macrophage activation is one of the important mechanisms of its action, and it has been reported that CD36 regulates LPS-induced ALI by enhancing macrophages M1 polarization (Sun et al., 2022). Similarly, Protein phosphatase 2A (PP2A) (He et al., 2019), sphingosine-1-phosphate (S1P) (Joshi et al., 2020), oxidative stress-induced FABP5-S glutathione acylation inhibits macrophage inflammation (Guo et al., 2021).

Neutrophils are crucial immune cells in ALI, and the regulation of neutrophils has been studied more and more. Targeted binding adhesion molecule c can improve ALI caused by sepsis by reducing CXCR4^+^ neutrophils (Hirano et al., 2018). In addition, PDL1 and GM-CSF also alleviated the course of ALI by promoting the completion of neutrophil traps (De Alessandris et al., 2019; Zhu et al., 2022).

**FIGURE 2:**
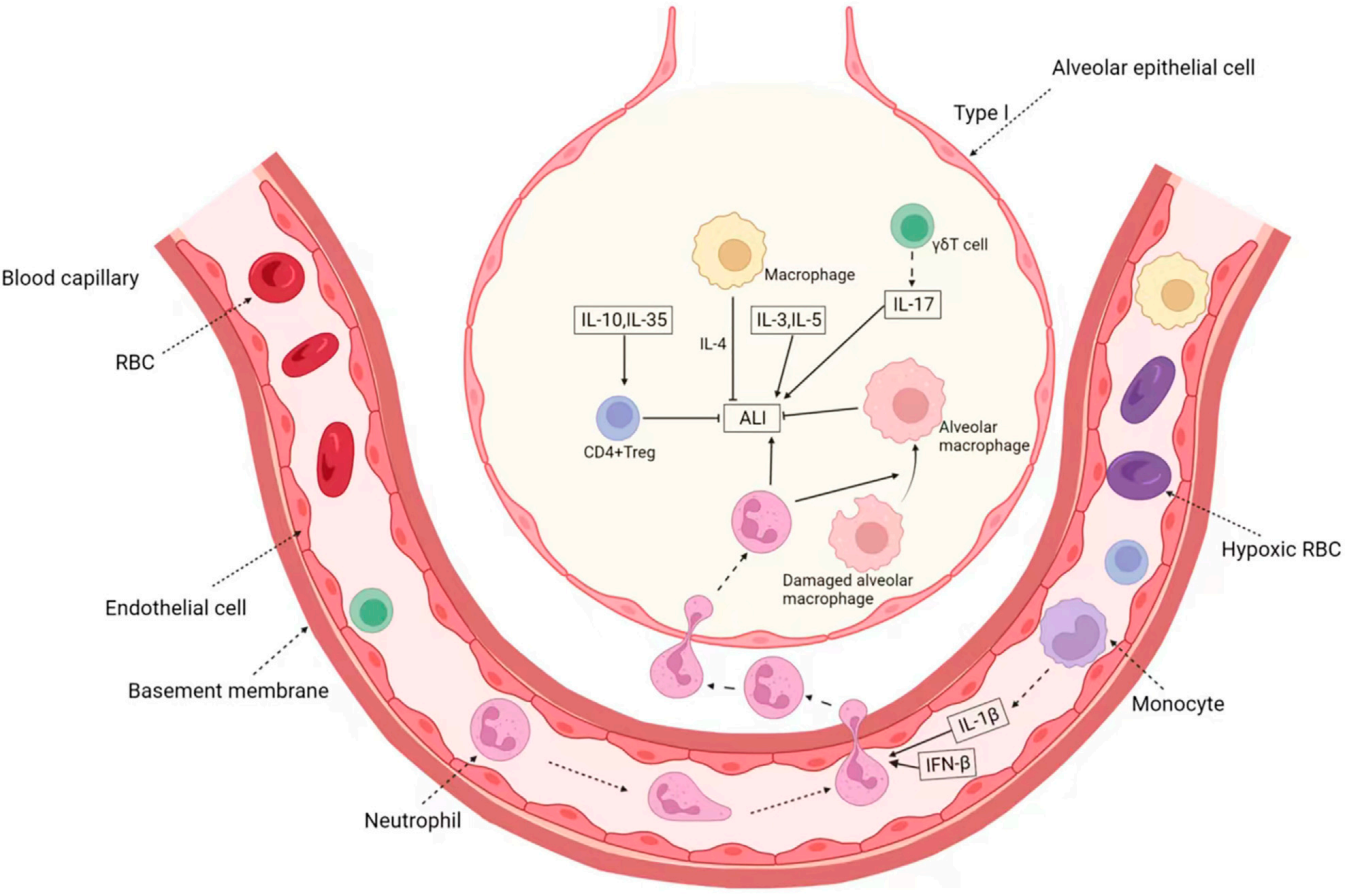
The Immune-related factors in ALI/ARDS. In ALI, immune cytokines mainly play the role of cell communication, which is represented by interleukin. Anti-inflammatory cytokines IL-10 and IL-35 can induce the activation of CD4^+^ Treg and then inhibit ALI. IL-1β produced by monocyte and IFN-β can induce the activation of neutrophil, which aggravate ALI. Besides the factors secreted by recruited immune cell, IL-3 and IL-5 can directly promote ALI. On the other hand, activated γδT cells and macrophages can secrete IL-17, IL-5 and so on, forming positive feedback. In addition, immune cells represented by M2-type macrophages can also secrete IL-4 to alleviate the progression of inflammation.

In other cells, for example, overexpression of cAMP response element modulator (CREM) in T cells exacerbates lipopolysaccharide-induced ALI (Verjans et al., 2013), and NKG2D-activated natural killer cells mediate lung ischemia-reperfusion injury (Calabrese et al., 2021). We summarized the role and mechanism of immune cytokines in ALI as shown in the Figure 2.

## 6 Prospects for future treatment strategies for ALI

As a disease with rapid onset and rapid course, there is no specific treatment strategy for ALI. Recently there has been a growing focus on the critical role of immune cells and immune-related factors in various diseases, including tumors. The progress has been enormous and valuable. As ALI is a disease with uncontrolled immune cells and inflammatory storms, the application of immunotherapy will have a high prospect. Just as a study by Xu et al. (2020) recently, they retrospectively studied 21 patients with severe and critical COVID-19 who were treated with tocilizumab, an IL6 receptor inhibitor. Surprisingly, their results showed that a majority of patients with tocilizumab experienced an immediate improvement in both symptoms, hypoxygenemia, and CT opacity changes after treatment. As a result, they concluded that tocilizumab improves clinical symptoms and inhibits deterioration in severe COVID-19 patients, which is an effective treatment option for COVID-19 and can provide a therapeutic strategy for this deadly infectious disease. Furthermore, their team also identified a monocyte subpopulation that promotes the inflammatory cytokine storms by using single-cell mRNA sequencing in two severe-stage COVID-19 patients before and after tocilizumab treatment. In spite of the fact that tocilizumab treatment reduces inflammation, immune cells (including plasma B cells and CD8^+^ T cells) still exert a robust antiviral response both humorally and cellularly. Therefore, they indicated that treatment with tocilizumab can not only reduce the damage caused by monocyte inflammatory factor storms, but also maintain the normal antiviral immune response of COVID-19 (Guo et al., 2020).

However, despite the promise of immunotherapy, few drugs are available for clinical use. The use of monoclonal antibodies or antagonists to neutralize cytokines such as TNF, IL-1, and IL-8 can significantly reduce lung injury in animal studies, but most clinical trials have negative results. The results of clinical trials showed that anti-TNF monoclonal antibody (Afelimomab) was used to treat severely infected ALI, among which the MONARCS study (*n* = 2,634) showed that the mortality of the Afelimomab treatment group was significantly reduced in severely infected patients with high or low levels of IL-6 (Panacek et al., 2004; Rondon and Venkataraman, 2005). But another study did not reduce the case fatality rate (Reinhart et al., 2001). There is a lack of clinical evidence on whether cytokine monoclonal antibodies or antagonists can be used in the treatment of ALI/ARDS. Moreover, whether Itolizumab, a novel anti-CD6 monoclonal antibody, can be used in ARDS caused by COVID-19 has also attracted wide attention. On the one hand, previous clinical data showed that Itolizumab could significantly reduce IL-6 level in patients with psoriasis and internal rheumatoid arthritis (Budamakuntla et al., 2015; Aira et al., 2016); On the other hand, data also showed that patients with COVID-19 experienced severe second-degree heart block after infusion of Itolizumab (Kumar et al., 2021). Therefore, judging from the current research and clinical data, there is still a lot of work to be done on immunotherapy for ALI in the future. It is important to fully understand the immunological pathogenesis of ALI and regulate the immune balance, rather than simply regulate a certain inflammatory factor. Drugs that target a specific cytokine are only capable of inhibiting that cytokine, and may not limit the effects of other cytokines as well. As a consequence, selecting the correct time period for anti-inflammatory therapy and identifying those patients who would benefit from immunosuppression remains crucial (Mastitskaya et al., 2021).

Although we have encountered some setbacks in the process of applying immunotherapy to ALI, there is no denying that the initial exploration of immunotherapy has brought new hope to more patients. The immune mechanism of ALI is very complex. Multiple immune cells interact with each other and play different roles. Monoclonal antibody, as a key drug in immunotherapy, is always accompanied by serious side effects in the process of immunotherapy, which makes it unable to play its proper therapeutic role. On the one hand, this is due to our insufficient understanding of the immune pathogenesis of ALI, and on the other hand, it is also caused by the versatility of the drug targets of monoclonal antibody therapy. Perhaps, the screening and study of compounds that target the immune system is a meaningful therapeutic strategy, and the use of combination medication regimen to prevent side effects is also a viable approach.

In a word, for the immunotherapy of ALI, it is difficult but indispensable to maintain the dynamic balance of various immune cells. Perhaps a good strategy is to have a deeper understanding of the immune cytokines concerned with the development of disease, this opens up new therapeutic targets for ALI.
